# Spatiotemporal Characteristics and Patterns of the COVID-19 Pandemic in China: An Empirical Study Based on 413 Cities or Regions

**DOI:** 10.3390/ijerph19042070

**Published:** 2022-02-12

**Authors:** Jialu Shi, Xuan Wang, Fuyi Ci, Kai Liu

**Affiliations:** 1 College of Geography and Environment, Shandong Normal University, Jinan 250358, China; jialusunny@126.com (J.S.); h762442@163.com (X.W.); 2 School of Economics, Shandong Normal University, Jinan 250358, China; fuyici@163.com

**Keywords:** COVID-19, pandemic analysis, spatiotemporal distribution, spatiotemporal patterns, China

## Abstract

The global economy was stagnant and even regressed since the outbreak of COVID-19. Exploring the spatiotemporal characteristics and patterns of COVID-19 pandemic spread may contribute to more scientific and effective pandemic prevention and control. This paper attempts to investigate the spatiotemporal characteristics in cumulative confirmed COVID-19 cases, mortality, and cure rate in 413 Chinese cities or regions using the data officially disclosed by the government. The results showed that: (1) The pandemic development can be divided into five stages: early stage (sustained growth), early mid-stage (accelerated growth), mid-stage (rapid growth), late mid-stage (slow growth), and late-stage (stable disappearance); (2) the cumulative number of confirmed COVID-19 cases remained constant in Wuhan, whilst the mortality tended to rise faster from the early stage to the late-stage and the cure rate moved from the southeast to the northwest; (3) the three indicators mentioned above showed significant and positive spatial correlation. Moran’s *I* curve demonstrated an inverted “V” trend in cumulative confirmed COVID-19 cases; the mortality curve was generally flat; the cure rate curve tended to rise. There are apparent differences in the local spatial autocorrelation pattern of the three primary indicators.

## 1. Introduction

COVID-19 has by far spread to 214 countries on six continents, with more than 280 million confirmed cases. The pandemic is particularly severe in Europe and North America. The United States, India, Brazil, the United Kingdom, Russia, Turkey, France, Germany, Iran, and Spain account for the top ten in the cumulative number of confirmed cases. (https://ncov.dxy.cn/ncovh5/view/pneumonia?clicktime=1579579384&enterid=1579579384&from=timeline&isappinstalled=0&scene=2, access on: 27 December 2021) Worse still, the cumulative number of confirmed COVID-19 cases remains on the rise worldwide. However, the joint effort to prevent and control the pandemic is greatly hindered by global instability, political systems, rulers’ negligence, and pandemic prevention policies. Population and trade flow in the context of globalization also facilitates the flow of confirmed cases between countries. The mutation of the virus poses a huge challenge to the medical care system, and shortages of medical equipment and facilities still linger. Pandemic prevention and control remain a considerable challenge for the entire world. The world economy, impacted by the pandemic, plunges into a downturn, which is complicated by chaotic social governance and people’s panic far beyond the expectations of various countries. During the global economic crisis starting from 2008, the GDP growth rate of the United States in the second quarter of 2009 decreased by 3.92% year-on-year, the lowest in the five years around the period. In contrast, the US GDP growth rate dropped by −9.03% on a year-on-year basis in the second quarter of 2020, a shocking economic setback (https://data.cnki.net/ access on: 20 December 2021). In March 2020, the International Olympic Committee postponed the Tokyo Olympics for a year due to the COVID-19 pandemic. (http://www.xinhuanet.com/2021-03/23/c_1127245963.htm access on: 10 December 2021) The games were canceled only three times due to war in its long history, and this was the first time that it was unscheduled for reasons other than war. In addition to the disrupted economic markets and social and cultural life, political activities in Europe, such as elections in 2020, were also affected. (http://www.xinhuanet.com/world/2020-03/16/c_1125719311.htm access on: 16 December 2021) (http://www.xinhuanet.com/world/2020-08/03/c_1126320854.htm access on: 16 December 2021) Right-wing populists in many European countries took the opportunity to stir up the “Eurosceptic” wave, and European integration was again pushed to a “crossroads”. To sum up, the pandemic has impacted the whole world profoundly and hindered the healthy development of the world substantially in all aspects.

Unidentified COVID-19 cases were first found in December 2019 in Wuhan, China. In January 2020, the World Health Organization (WHO) named this viral pneumonia “COVID-19” and characterized it as a “Public Health Emergency of International Concern”. As of 26 December 2021, the confirmed COVID-19 cases in China accumulated 24.5 times that of SARS in 2003. China witnessed an early outbreak of COVID-19, and its dense population and high mobility make the prevention and control of COVID-19 more challenging than ever. 

This paper is presented in five parts. The first part presents the development status of the COVID-19 pandemic as well as its harm and impact. The second part reviews the existing literature in the early stage and the late mid-stage. The third part describes the data and methods used for the study. This paper presents an empirical study on 413 Chinese cities or regions that reported their pandemic data regarding cumulative confirmed COVID-19 cases, mortality, and cure rate from 20 January to 19 March 2020. The fourth part presents the empirical study. In this part, the daily number of newly confirmed cases was taken as the key criterion, and the mortality rate and cure rate were included to divide the stages of pandemic spread and identify the characteristics of each stage. Meanwhile, the trend of the pandemic spread was analyzed. Then, the center of gravity model was used to calculate cumulative confirmed cases, mortality, and cure rate, and ArcGIS was employed to visualize the evolutionary trajectory of the three indicators. Then, the spatial autocorrelation model was adopted to identify the spatiotemporal pattern of cumulative confirmed COVID-19 cases, mortality, and cure rate. The global autocorrelation model was utilized to determine the trend of the global correlation of the three indicators. The local autocorrelation model was applied to delve into the spatial characteristics of the three indicators, and ArcGIS was used to visualize the representative fragments in the research period. The last part summarizes the main conclusions of the study.

## 2. Literature Review

The COVID-19 pandemic has significantly impacted the socioeconomic development in China and many other countries in the world. Therefore, scholars have made substantial efforts to explore ways to alleviate the pandemic and offer suggestions from various perspectives. 

In general, the research topics for the early stage COVID-19 pandemic mainly focus on comprehensive prevention and control of the pandemic [1,2,3], pandemic risk assessment [3,4,5], spatial diffusion process of COVID-19 cases [4,5,6,7,8,9], temporal evolution characteristics [4,5,6,7,9], the impact of population migration on the pandemic [10,11], application of big data [12,13], and management of public opinions [14,15]. Wang et al. [8] found that the COVID-19 pandemic showed geographical patterns such as proximity diffusion, hierarchical diffusion, migration diffusion, and corridor diffusion in space. Li et al. [9] believed that in terms of temporal evolution, the pandemic began from Major Snow, raged at the Start of Spring, declined in the Awakening of Insects, and lagged in the Vernal Equinox. Based on the big data of migration from Baidu, Tong et al. [10] concluded that the pandemic impacted migration after the Spring Festival. The pandemic had a noticeable impact on the communication activities between cities on weekends and holidays and regional differences. Xue et al. [12] showed that geography made positive contributions to the early prevention and control of the pandemic and that big data and the new generation of technical methods played a supporting role in preventing and controlling the pandemic. Through tracking analysis, Wang et al. [15] concluded that the overall response of the Chinese public to COVID-19 was rational and cheerful, but the spatial distribution of various topics was significantly different within the region. 

In the late mid-stage of the pandemic, researchers began to scrutinize the impact of COVID-19 on the economy, society, politics, culture, ecology, and other related aspects.

The economic impact of COVID-19 is pervasive. Research topics to this end mainly involves its impact on economic development [16], economic globalization [17], input-output and supply chain [18,19], fiscal policy [20], industrial transformation [21], resource allocation [22], research cooperation [23], “agriculture, rural areas, and rural residents” [24], digital economy [25,26], private economy [27], income of vulnerable groups [28], and mass consumption [29]. Liu et al. [16] believed that the pandemic slowed down expanding regional differences in China and aggravated the imbalance within the region. Liu’s study [17] revealed that the COVID-19 pandemic could not affect the capital and technology drives of globalization but could affect the openness of countries. Liu et al. [18] analyzed the economic impact path of COVID-19 based on the input–output framework, and the results demonstrated that Provinces with a high degree of economic dependence on Hubei Province faced a greater economic impact, while Hubei Province, as the center of the pandemic in China, was expected to pay special attention to the agriculture, transportation, and construction industry. Ino et al. [19] analyzed how the pandemic has impacted the Global Supply Chain (GSC) and the proper approach to Supply Chain Risk Management (SCRM) in the future. Zhu et al. [21] examined the industrial transformation of pandemic prevention materials in various cities in China during the pandemic period and found that the technology-related density of a city can better represent its industrial transformation capacity. Barrero et al. [22] believed that the COVID-19 was an impact on resource reallocation. They predicted that 42% of temporary work stoppages would turn into long-term unemployment, and such impact on resource reallocation could be prevented by taking measures such as unemployment benefits and government subsidies. Qi et al. [24] concluded that COVID-19 had an adverse impact on the breeding industry, vegetable and fruit industry, flower industry, aquaculture industry, and other agricultural industries. 

Sociological studies on the pandemic mainly focused on employment [30,31,32,33], followed by population mobility after returning to work [10,34], social security [35], and people’s psychological state [36]. Webb et al. [33] thought that to create fair, resilient, and ethical structures for workers, businesses, economies, and societies at large, explicit support shall be provided by the government for those in informal employment. In terms of politics, previous studies mainly tried to compare the difference between countries under the influence of systems and regional governance measures [37,38]. 

At the cultural level, previous research efforts were mainly devoted to the correlation between global security culture and the pandemic [39], spatial reconstruction of regional cultural consumption [40], and the influence on culture during the pandemic [41]. In terms of ecology, the existing studies mainly examined the ecological environment emergency management in the context of the pandemic [42,43] and the impact of different environments on the pandemic [44,45,46]. Dong et al. [42] believed that, in the face of major pandemics, the environmental emergency response mechanism should be improved, the basic capacity building of environmental emergency response should be strengthened, and the support and guarantee system should be enhanced. Smit et al. [44] found that the evidence elicited so far suggests a weak modulation effect, which is overwhelmed by the transmission scale and rate of COVID-19. If there is seasonally modulated transmission, it will become more apparent in 2021 and the years to come. Azuma et al. [46] showed that the increase of the pandemic was substantially correlated with the increase of daily temperature or sunshine hours, and the increase of interpersonal contact caused by more outdoor activities on warm or sunny days may enable a faster spread of the pandemic. Ward et al. [47] indicated that COVID-19 cases hiked in the period of low relative humidity. 

There are extensive studies on cumulative confirmed COVID-19 cases at various scales, but comprehensive studies based on the cumulative number of confirmed cases, mortality rate, and cure rate are still lacking. The analysis of the temporal characteristics and dynamic spatial distribution of pandemic development in the whole city or region at the national level based on the three indicators need to be further improved, and the trajectory of the shift of the center of gravity is rarely studied. 

Hence, this paper explores the trajectory of the center of gravity and spatial correlation of the three significant indicators (cumulative confirmed COVID-19 cases, mortality, and cure rate) in 413 cities or regions with pandemic reports in China. The contribution of this study is: Exploring the spatiotemporal distribution characteristics and patterns may provide some scientific ideas for the analysis and summary of the first wave of COVID-19 in China, as well as for the prevention and control of small-scale outbreaks of COVID-19 ever since 19 March 2020. It will also be instrumental in improving the research system of COVID-19 and providing a reference for other countries suffering from the COVID-19 pandemic around the world.

## 3. Data Source and Methods

### 3.1. Data Source

In this study, 413 cities or regions with reported pandemic data were studied, and the original data were derived from the official COVID-19 reports by the national, provincial, municipal, and district health commissions (http://www.nhc.gov.cn/ access on: 20 December 2021). Taking into account the administrative level of the municipality, each district of the municipality was considered an independent research unit. Hong Kong, Macao, and Taiwan were not taken into consideration in this study due to the lack of related data.

The research period for this study was from 20 January to 19 March 2020, a period when the pandemic spread rapidly and a crucial and necessary period for us to explore the spatiotemporal spread patterns of COVID-19. This study may facilitate future follow-up of pandemic prevention and control, policymaking in terms of case tracking, healthcare resource allocation, and recovery of production and life, provide essential data for people’s psychological counseling and vaccine delivery, and offer proper and precise guidance for the healthy development of the region in all rounds. Since the incubation period of COVID-19 is defined as 1–14 days according to the “COVID-19 Control Scheme (fourth edition)” issued by the National Health Commission of China, and the average latent period is 5.2 days (http://www.xinhuanet.com/2020-02/03/c_1125523740.htm access on: 26 May 2020), this study collected and analyzed the data of nine daily sections from 23 January 2020 (when Wuhan began to be locked down) to 19 March 2020 (the daily number of newly confirmed cases was 0 in a row for the first time), with a six-day interval. The data after this period were not included as the COVID-19 pandemic was generally stable in China. Although there were new cases in Beijing, Hebei, Xinjiang, the three Provinces in the Northeast of China, Zhejiang, Shanxi, and so forth, the daily number of newly confirmed cases was under 200, which did not show prominent spatial characteristics.

### 3.2. Methods

#### 3.2.1. Center of Gravity Model

The center of gravity is a physical concept, but it can also express the overall spatiotemporal evolution of a particular regional phenomenon [48]. In this study, the model was mainly used to analyze the spatial difference and evolution of cumulative confirmed COVID-19 cases, mortality, and cure rate in different regions in China. The calculation formula is as follows:

(1) $$X=\frac{\sum_{i=1}^{n}x_{i}a_{i}}{\sum_{i=1}^{n}a_{i}},\;Y=\frac{\sum_{i=1}^{n}y_{i}a_{i}}{\sum_{i=1}^{n}a_{i}},\;$$

In the formula, *X* and *Y* are coordinates of the center of gravity in terms of the spatial distribution of cumulative confirmed COVID-19 cases, mortality, and cure rate in different regions in China. $a_{i}$ represents the number or proportion of cases in region *i*. $x_{i}$ and $y_{i}$ are the central geometric coordinates of municipal administrative unit *i*. *n* is the number of municipal administrative units.

#### 3.2.2. Global Spatial Autocorrelation

In the present study, global spatial autocorrelation was utilized to reflect the spatial agglomeration of cumulative confirmed COVID-19 cases, mortality rate, and cure rate of COVID-19 in different regions of China. The global Moran’s *I* is calculated as follows:

(2) $$Moran’s\;I=\frac{n\sum_{i=1}^{n}\sum_{i=1}^{n}W_{ij}\left(x_{i}-\bar{x}\right)\left(x_{j}-\bar{x}\right)}{(\sum_{i=1}^{n}\sum_{i=1}^{n}W_{ij})\sum_{i=1}^{n}{\left(x_{i}-\bar{x}\right)}^{2}},\;(i\neq j)$$

In the formula, $x_{i}$ and $x_{j}$ are the cumulative confirmed COVID-19 cases, mortality, and cure rate on sample sites *i* and *j*. $\bar{x}$ is the mean value of all observed values in the study area. *n* represents the number of samples. $W_{ij}$ is the spatial weight [49]. The global Moran’s *I* is between [−1, 1]. A Moran’s *I* > 0 indicates that there is a positive correlation between the number or ratio of cases in space, while a Moran’s *I* < 0 suggests that the number or ratio of cases is negatively correlated in space. When Moran’s *I* is equal to 0, it indicates no spatial autocorrelation between the number or ratio of cases.

#### 3.2.3. Local Spatial Autocorrelation

Local Moran’s *I* was employed to represent the degree of regional spatial agglomeration better and reveal the correlation between spatial reference units and their neighboring units [50]:

(3) $$Local\;Moran’s\;I=\frac{n\left(x_{i}-\bar{x}\right)\sum_{j=1}^{m}W_{ij}\left(x_{j}-\bar{x}\right)}{\sum_{i=1}^{n}{\left(x_{i}-\bar{x}\right)}^{2}}$$

In the formula, $x_{i}$ and $x_{j}$ are the cumulative number of confirmed cases, mortality, or cure rate on sample sites *i* and *j*. $\bar{x}$ represents the mean value of all observed values in the study area. *n* is the number of samples. $W_{ij}$ is the spatial weight.

## 4. Research Results

### 4.1. Stage Characteristics of Pandemic Spread

The actual development of the COVID-19 pandemic in China was divided into five stages (Table 1), taking the daily number of newly confirmed COVID-19 cases as a critical criterion and including the mortality and cure rate in the study.

In the early stage, the frequent travel of migrant workers returning to their hometown during the Spring Festival made it possible for the pandemic to spread quickly in the provinces closely related to Hubei, such as Henan, Guangdong, and Zhejiang. In this stage, the overall mortality and cure rates fluctuated between 2% and 5%. In the early mid-stage, the number of newly confirmed cases on 27 January exceeded 1000 for the first time, and the growth rate reached over 100% for the first time since statistics (130%). The number of newly confirmed cases started to leap by thousands on a daily basis and reached a peak on 4 February (according to the initial detection standard). On 1 February, the cure rate began to surpass mortality, and the gap between them doubled since then. In the mid-stage, on 12 February, clinical tests began to be included in the diagnosis, and more comprehensive and rigorous testing methods led to an abnormal surge of newly confirmed cases on 12 and 13 February, but the situation was brought under control soon. In this stage, the mortality rate and cure rate rose gradually, but the cure rate increased more obviously. In the late mid-stage, on 19 February, the number of newly confirmed cases dropped to 100 and below. On 5 March, the number of newly confirmed cases outside Hubei Province reached 0 for the first time, showing the effectiveness of the “lockdown”. During this period, the increase of mortality and cure rates was first large and then small, and the turning point of the increase of mortality rates came earlier. In the late-stage, the daily number of newly confirmed cases nationwide fell off to a double-digit number. On 18 March, the number of newly confirmed cases reached 0 for the first time in Hubei Province. The mortality and cure rates across the country remained stable at their respective high levels. Specifically, the mortality rate hovered at 4%, and the cure rate went up to over 90%. The pandemic was brought under effective control around the country; the resumption of work and school activities were carried out in an orderly manner; economic and social activities were running normally.

**Figure 1:**
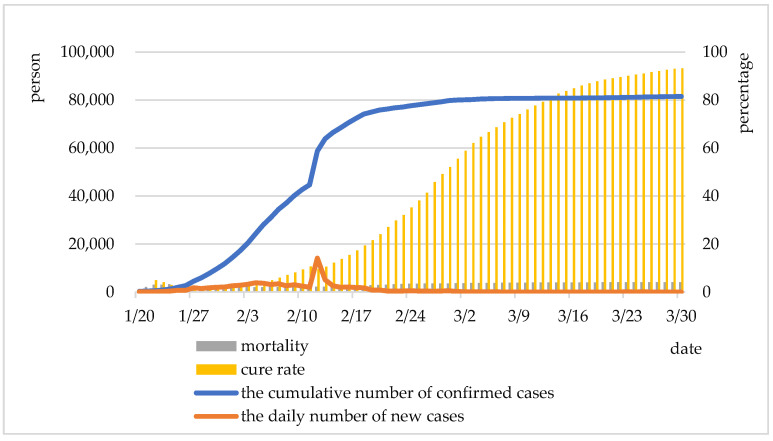
The transmission trend of COVID-19 in China.

Figure 1 demonstrates that the cure rate was the smoothest temporally. The earliest turning point of the three curves appeared in the mortality rate (27 January), mainly due to the low patient base, lack of clinical experience, and high mortality rate. Influenced by the change of clinical testing standards, the three curves showed the second turning point: The cumulative number of confirmed COVID-19 cases surged; the mortality increased, while the cure rate decreased. This also shows the importance of clinical testing standards. The reason why the peak value is not ideal this time is for a sounder treatment system, which is reflected in the rapid improvement and more stable convergence after the peak value. The insignificant turning point of the cure rate on February 18 and the mortality on 24 February indicated that the rise tended to slow down as the source of the pandemic was effectively controlled.

### 4.2. Change Characteristics of the Pandemic Center of Gravity

**Figure 2:**
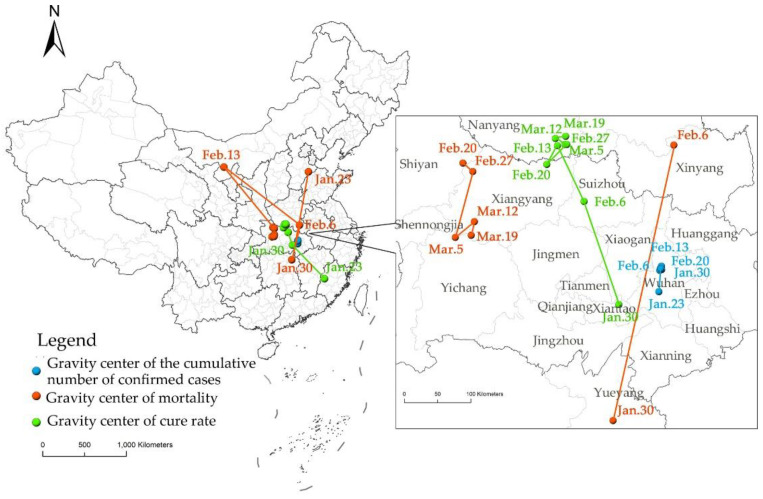
The center of gravity of COVID-19 in China.

The temporal changes of cumulative confirmed COVID-19 cases, mortality, and cure rate reflect the dynamics in the spatial distribution of COVID-19 across the country. According to the calculation results of the pandemic center of gravity center (Figure 2), there was no significant change in the spatial regularity of the distribution of the cumulative confirmed COVID-19 cases in China during the research period. The center of gravity remained in Wuhan, the first place where the pandemic was discovered in China (geographical coordinates: 114.23° E–114.31° E, 30.49° N–30.82° N).

In contrast, the center of gravity in mortality across the country shifted markedly over time. It experienced a remarkable migration (Cangzhou City, Yueyang City, Xinyang City, Alxa League) through the early stage to the mid-stage of the pandemic, and the maximum distance was up to 1139.18 km. In the late mid-stage, the center of gravity began to remain within Hubei Province (Shiyan City, Xiangyang City, Yichang City, Xiangyang City). This phenomenon was closely related to the cumulative confirmed COVID-19 cases and deaths in different regions: The early stage and mid-stage were the initial stages of the pandemic where most areas had confirmed cases, but no deaths, and therefore the center of gravity was dominated by a small number of areas with non-zero mortality rates. Affected by the base number, Wuhan city featuring the largest number of cumulative confirmed cases may not have the highest mortality, while regions far away from Wuhan city with a small number of cumulative confirmed cases may witness a high mortality rate due to “the butterfly effect”. For example, the mortality in Cangzhou City reached 100% on 23 January, which was diluted by the increase in confirmed cases after that. The number of infected cases was minimal in Chengmai County in Hainan Province on 30 January and Shihezi City in Xinjiang Province on 13 February, but deaths were almost equal. Therefore, the center of gravity regarding mortality rates in this period was not located in Wuhan but moved in Hebei Province and Inner Mongolia Autonomous Region.

In terms of cure rates, the center of gravity moved spatially from southeast to northwest roughly. In the early stage, the center of gravity was located in Sanming City of Fujian Province, then moved to Xiantao City and Suizhou City of Hubei Province in the early mid-stage, and then remained in Nanyang City of Henan Province after the mid-stage. The only rebound (on 20 February) was in neighboring Xiangfan City of Hubei Province. The mechanism of large migration in the cure rate in the early stage was the same as that of the mortality. The center of gravity shifted from Fujian Province to Henan Province with a distance of 799.82 km, indicating that the cure rate increased rapidly in Northwest China, and the difference in cure rate between Southeast and Northwest China widened. After the mid-stage, the cure rate in some areas gradually rose to 100%; the center of gravity tended to be stable; and the cure rate tended to be balanced.

The centers of gravity of the three indicators did not involve the northwest, southwest, northeast, and southeast regions. The common feature of these regions was that they were far from the center of the pandemic, suggesting that the pandemic spread was attenuated by distance. 

### 4.3. Global Spatial Autocorrelation Characteristics of the Pandemic

**Figure 3:**
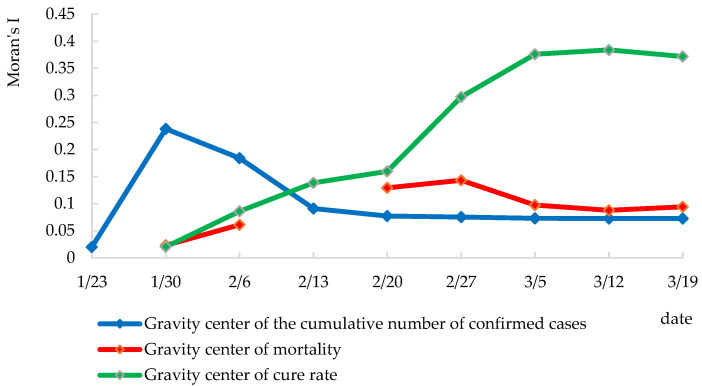
The global spatial autocorrelation of COVID-19 in China.

The global spatial autocorrelation Moran’s *I* of cumulative confirmed COVID-19 cases, mortality, and cure rate in each region was calculated, and the line chart of the indicators’ temporal variation trend was drawn (Figure 3).

Global Moran’s *I* reflects the general characteristics of the distribution of COVID-19 cases across the country. Throughout the research period, the Moran’s *I* of cumulative confirmed COVID-19 cases was >0, with positive values <0.01 in the *Z* test and *p*-value, passing the 1% significance test. The result revealed that the distribution of the cumulative confirmed COVID-19 cases in China showed positive autocorrelation in space. The cumulative number of confirmed cases displayed spatial agglomeration embodied in that areas with more confirmed cases cumulatively were closed to each other, or areas with less confirmed cases cumulatively were close to each other. 

Overall, the global Moran’s *I* of cumulative confirmed cases showed a wave pattern, with a low degree of spatial agglomeration in the early stage, a noticeable trend of spatial agglomeration in the early mid-stage, and an agglomeration peak on 30 January. It returned to fluctuation at a level in the mid-stage, and the decline weakened and tended to stabilize. At this point, the spatial correlation of cumulative confirmed cases decreased, which tended to disperse in space with lessening agglomeration. As millions of people who once lived and stayed in Wuhan dispersed to other areas in the early period of the Spring Festival travel rush, some of whom were COVID-19 carriers, the population movement accelerated the spread of the virus. The Spring Festival is the most important occasion for family reunions in China, and the COVID-19 pandemic occurred during this particular period, which further increased the possibility of the local spread of the virus. At the State Council press conference for joint prevention and control of COVID-19 on 11 February, Wu Zunyou, chief epidemiologist of the Chinese Center for Disease Control and Prevention, confirmed that family clusters accounted for 83% of the total number of clustered outbreaks (https://politics.gmw.cn/2020-02/11/content_33545700.htm access on: 12 October 2021). The spread route was usually that first-generation cases (first-generation cases refer to the first case who usually has a history of work, residence, and travel in Hubei province or Wuhan) led to the second-generation spread (second-generation cases refer to the second-generation transmission caused by family contact or dinner gatherings with the first case). The cases of the third and fourth generations were mainly caused by the fact that the clinical symptoms of the first-generation cases were not significant, and they lacked the prevention awareness when contacting these cases (http://www.gov.cn/xinwen/gwylflkjz09/index.htm access on: 2 October 2021). Generally, the second-generation cases had the highest infection rate, accounting for 64%, followed by 22% for first-generation cases, and third-generation and fourth-generation cases were in the minority. 

The Moran’s *I* curve of cumulative confirmed cases was generally in an inverted “V” pattern, indicating that the spatial autocorrelation of each region changed from weak to strong and then returned to weak. The differences of scattered points reached the peak in the mid-stage and early stage, while the agglomeration characteristics changed little in the mid-stage and late-stage. In the early mid-stage, the first-generation cases passed the virus latency, and the second-generation cases witnessed a concentrated outbreak. The regional agglomeration was unprecedentedly strong because of the close connection with the first-generation cases. With the extended measures such as regional restriction on population activities, the agglomeration weakened and became stable after the mid-stage. In the late mid-stage, the cumulative confirmed cases were relatively scattered in the whole country, retrospectively.

During the research period, the global spatial autocorrelation of mortality rates on 23 January and 13 February did not pass the significance test, while the Moran’s *I* of mortality rates at the other time points was more significant than 0, and *Z* test values were positive. The *p* values on 30 January and 6 February were less than 0.05, which passed the 5% significance test; while the *p* values at the other time points were less than 0.01, which passed the 1% significance test. During the research period, the Moran’s *I* of mortality rates was generally low and fluctuating and slightly higher in the late mid-stage. The mortality showed a weak agglomeration and scattered across the country, suggesting no second severe disaster area like Wuhan or Hubei during the whole pandemic prevention and control period.

The global spatial autocorrelation of cure rates on 23 January did not pass the significance test, which was related to the small number of cured cases. Most cases were still at the beginning or the onset stage. After that, the Moran’s *I* of cure rates was all greater than 0, with positive *Z* test values and *p* values less than 0.01, passing the 1% significance test. The Moran’s *I* of cure rates was generally rising, indicating that the cure rate increasingly agglomerated across the country. While the total number of confirmed cases of COVID-19 increased over time, the trend was slowed down by increasingly accurate clinical treatment, proper medical care, and enhanced pandemic prevention and control measures, resulting in a significantly lower mortality rate and a higher discharge rate. The continuous increase in cured cases across the country enabled each region to show a more substantial spatial agglomeration.

### 4.4. Local Spatial Autocorrelation Characteristics of the Pandemic

Lisa plots were used to identify the clustering types of cumulative confirmed cases, mortality rates, and cure rates of COVID-19 in different regions at different periods.

**Figure 4:**
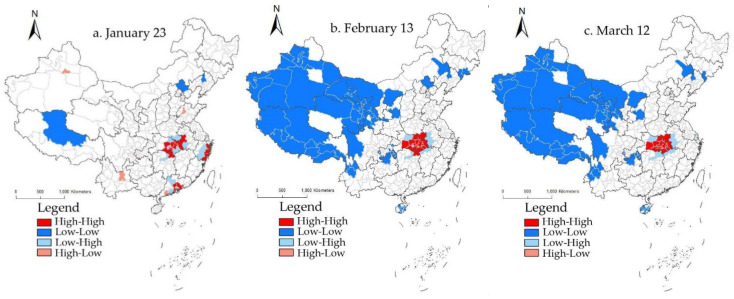
The Lisa plots of cumulative confirmed COVID-19 cases in China.

According to the Lisa plots of cumulative confirmed cases in the selected nine-day segments (Figure 4), there were not many regions with a correlation of cumulative confirmed cases during the research period. Except for 23 January in the early stage, the agglomeration characteristics of the accumulative confirmed cases in other day segments showed little difference, and especially after 27 February, the spatial agglomeration characteristics were utterly consistent. 

In the early stage, the high-high agglomeration was concentrated in Hubei Province, the eastern part of Zhejiang Province, and the Pearl River Delta urban agglomeration. These three regions had many cumulative confirmed cases and accounted for the gathering centers in the early stage of the COVID-19 pandemic. At the same time, the degree of radiation in these three regions to the surrounding areas was more robust, and the spatial differences within the hot spots were minor. High-low agglomeration was mainly distributed in Jinan and Kunming, where the cumulative number of confirmed cases was significant, but the number was small in the surrounding areas, resulting in a significant spatial difference between the two, mainly because the provincial capital cities could attract population from other places, and the high population flow speeded up the transmission of the virus. As the first place of the COVID-19 outbreak, Hubei Province did not show vast agglomeration of hot spots on 23 January, which resulted from the lengthy early diagnosis, a severe shortage of nucleic acid testing kits, and insufficient medical staff, equipment, and space, hence the limited number of confirmed cases on a daily basis. Therefore, during this period, the cumulative total of confirmed cases in Hubei Province reflected only the detection ability rather than the actual outbreak. Low-high agglomeration cities were distributed in the periphery of high–high agglomeration, where there were relatively few cumulative confirmed cases and they were surrounded by areas with high cumulative confirmed cases, accounting for a big difference between the two. Low–low agglomeration was distributed sporadically and located in the far western and northeastern regions. 

There was a dramatic change in the spatial distribution of the cumulative confirmed cases across the country on 30 January, and this spatial distribution continued until the late-stage. High–high agglomeration remained in Hubei Province, with increasing cities and fixed spatial locations. It accounted for the core area of the COVID-19 pandemic, and the radiation effect of this area on surrounding areas was increasing day by day. Hubei Province, the first place for the COVID-19 outbreak, had a large base of the infected population. In addition, frequent population migration within the province in the early stage expanded the spread range of the virus, and the problem of “data siltation” caused by inadequate detection capacity was solved continuously. This region remained the center of the COVID-19 pandemic. Since the COVID-19 outbreak, the efforts of the whole country were mobilized for pandemic prevention and control. Experts, medical personnel, and goods were allocated to Wuhan, which played an essential part in treating patients and cutting off the spread of the pandemic. Since 23 January, the “lockdown” measures around the cities of Hubei Province effectively blocked COVID-19 viruses from spreading to other provinces on a large scale. As a result, the agglomeration of hot spots remained stable in the late mid-stage. Low–high agglomeration cities were less distributed, banding around the periphery of high–high agglomeration. Cumulative confirmed cases in this region differed significantly from those in surrounding areas, and the radiation range was limited. Low–low agglomeration was widely distributed in the northwest inland, where the accumulative number of confirmed cases was relatively small. The surrounding areas displayed a roughly consistent accumulative number of confirmed cases, mainly because these regions were too far from the pandemic center of Hubei Province, and less population flowed to these regions. The spatial distance hindered the population flow, thereby reducing the transmission of the virus from person to person. 

**Figure 5:**
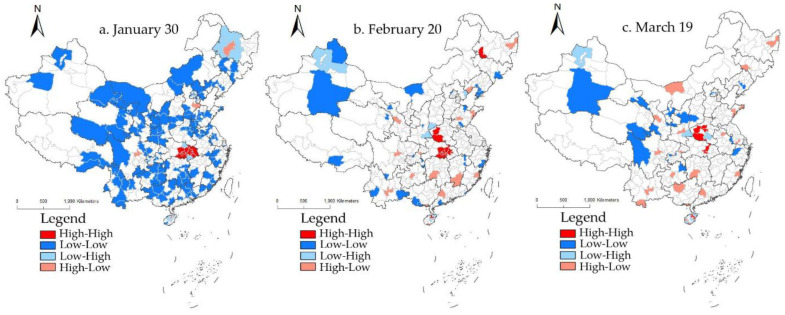
The Lisa plots of COVID-19 mortality in China.

Given the lag of mortality rates, part of the LISA plots (Figure 5) was presented by postponing the cumulative number of confirmed cases by one interval. Throughout the research period, the 7-day segments that passed the test showed a decrease in the area of spatial correlation. In the early mid-stage, low–low agglomeration was widely scattered in all Provinces except Hubei and Heilongjiang. High-high agglomeration was distributed in Wuhan, Huanggang, Xiaogan, and other areas in Hubei Province. High–low agglomeration and low–high agglomeration were scattered in the periphery or within hot spots and cold spots, and the mortality rate showed evident spatial autocorrelation. In the mid-stage, death cases of patients in the early stage began to appear intensively, resulting in a dramatic change in the spatial pattern of mortality. The “high–high” pattern in Hubei Province was prominent, and the cold spots were reduced to sporadic distribution due to the spread of the pandemic. Afterward, the spatial pattern remained stable. From the late mid-stage, the mortality hot spots spread from Wuhan in Hubei Province to Nanyang and Luoyang in Henan Province, where the situation was the most serious, and the hotspot center shifted to Henan Province in the late-stage. After the late mid-stage, the scope of mortality hot spots was reduced, and the radiation coverage in the surrounding areas was increasingly limited, indicating that the pandemic was effectively controlled. The number of low–low agglomeration areas continued to decrease, and their spatial locations changed. In the early mid-stage and mid-stage, the number of low–high and high–low agglomeration cities increased, while the spatial location of each region remained unchanged in the late mid-stage.

**Figure 6:**
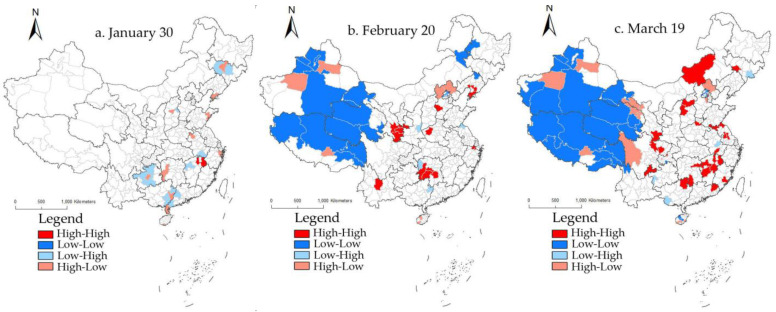
The Lisa plots of COVID-19 cure rates in China.

The same method was used to select the Lisa plots of cure rates (Figure 6). The 8-day segments that passed the test showed a changing spatial agglomeration of cure rates. The cold spots were distributed in large clumps in the west of “Heihe-Tengchong Line” and gradually expanded after the early mid-stage, mainly related to the low number of cases in the west. Since most of the patients were in the early stage of the pandemic, there were fewer deaths. Therefore, the spatial correlation of cure rates became increasingly prominent from the early mid-stage. In the meantime, high–low agglomeration was scattered around the cold spots, while low–high agglomeration was scattered around the cold spots or hot spots, showing a transition region as a whole. Hot spots were primarily distributed east of “Heihe-Tengchong Line” and gradually increased with the advent of the mid-stage and late-stage, reaching the peak in the late mid-stage and then decreasing. This was related to the rich and increasingly accurate clinical treatment experience and the effective prevention and control work of the whole country. Progress in medical and health technology increased the number of people discharged from hospitals, slowed the increase of cumulative confirmed cases, and improved the cure rate. 

Notably, the cure rate in the center of the outbreak in Hubei Province was not spatially correlated. The distribution of the cure rates had no regular patterns, but this did not show that the cure of COVID-19 in Hubei was unsatisfactory because the symptoms of confirmed cases in various cities of this province were more complex and challenging, as its number of cases accounted for 4/5 of the whole of China, the municipal cases symptoms the complexity and difficulty of higher population. 

## 5. Conclusions and Discussion

Based on the daily pandemic data released by health commissions at all levels, this paper presents an empirical study on the spatiotemporal characteristics and patterns in 413 Chinese cities or regions that reported their pandemic data in terms of the three indicators (cumulative confirmed COVID-19 cases, mortality, and cure rate). The main conclusions are as follows:

1. The COVID-19 pandemic in China from January to March 2020 is roughly divided into five stages with respective characteristics. The improvement in detection methods induces the turning points, which are conducive to the stable convergence of the pandemic in the later stage.

2. The center of gravity of each indicator demonstrates that the center of gravity in terms of cumulative confirmed cases remains in Wuhan; the center of gravity in terms of mortality rates leaps in the early stage and remains stable in Hubei Province in the late-stage and late mid-stage; the center of gravity in terms of cure rates moved from southeast to northwest, and finally stabilizes in Anyang City, Henan Province, on the border with Hubei Province.

3. The global Moran’s *I* of each indicator demonstrates a significant spatial positive correlation in cumulative confirmed cases, mortality, and cure rate. Overall, the Moran’s *I* of cumulative confirmed cases shows an inverted “V” pattern; the Moran’s *I* of mortality rates is stable, except that it is slightly higher in late mid-stage; the Moran’s *I* of cure rates continues to rise.

4. During the research period, few cities or regions have a spatial correlation in cumulative confirmed cases, and the spatial agglomeration characteristics are entirely consistent since the late mid-stage. The number of cities or regions with spatial correlation in mortality rates decreases; the scope of hot spots narrows; and the center of gravity of hot spots gradually moves north from Hubei to Henan Province. The spatial agglomeration of cure rate is constantly changing, and the number of cities with hot spots increases and becomes more and more discrete.

The spatiotemporal characteristics and patterns of the COVID-19 pandemic can help us intuitively feel the direction and development. From a general perspective, the pandemic was effectively controlled from the official response in January 2020 to late March. China has set a good example that effectively controls the pandemic. Its efficiency in patients’ treatment rate and the efforts to cut off the spread. The early decision of “lockdown” was correct and timely, which effectively delayed and reduced the time and scale of pandemic outbreaks in other cities. Since the mid-stage, the isolation of the pandemic in various regions became effective, and the high-risk areas were effectively controlled in Hubei Province. The gradual resumption of production and work in the later stage did not contribute to a new round of spread climax. This explorative effort enables the rapid and effective blocking of small-scale outbreaks of COVID-19 from 19 March 2020 onwards, and provides guidance for the daily performance of normalized and precise prevention and control as well as regionally differential combat against the pandemic. Various regions can reference it to formulate various resumption plans at different levels, thereby promoting dynamic pandemic prevention and control, restoring the production and life safely and effectively, and unfreezing the economy both adequately and rap rapidly.

Existing studies on the COVID-19 epidemic in China generally purport that geographic proximity and network proximity are important influencing factors for the spread of COVID-19 [4,7,8]. Clustered spread is also a main feature of the pandemic. For this reason, dynamic monitoring of population flow serves to block or reduce the risk of the spread of the COVID-19 pandemic. This study also confirms these conclusions.

Host behavior is one of the decisive factors in the dynamics of infectious disease transmission, and the COVID-19 pandemic is largely affected by the movement of the population. This paper systemically explores the spatiotemporal characteristics of the pandemic, without giving consideration to factors such as host behavior and population flow. Future research efforts may be devoted to simulating the spatiotemporal transmission of the pandemic by coupling up with the movement of population, in order to provide planning and analysis tools for pandemic prevention and control as well as public health research.

## Figures and Tables

**Table 1:** The stages of the COVID-19 pandemic in China.

Stage	Time	Characteristics
Early stage (sustained growth)	Before 26 January	The cumulative number of confirmed cases continued to grow, and the provinces with close contact with Hubei increased rapidly.
		The mortality fluctuated in an inverted “U” pattern.
		The cure rate decreased at a low level.
Early mid-stage (accelerated growth)	27 January–11 February	The cumulative number of confirmed COVID-19 cases increased remarkably at a high level. The daily number of newly confirmed cases reached its first peak and increased nationwide.
		The mortality fluctuated at a low level.
		The cure rate rose at a low level.
Mid-stage (rapid growth)	12 February–18 February	The daily number of newly confirmed cases witnessed the second peak and then dropped stably. The cumulative number of confirmed COVID-19 cases was generally high with a slower increase.
		The mortality rose rapidly.
		The cure rate rose rapidly.
Late mid-stage (slow growth)	19 February–5 March	The daily number of newly confirmed cases decreased gradually, and the number in Provinces other than Hubei was 0.
		The mortality continued to increase rapidly first and slowly then.
		The cure rate continued to increase rapidly first, slowly then, and stably finally.
Late-stage (stable disappearance)	After 6 March	The daily number of newly confirmed cases slowed down and declined gradually to 0.
		The mortality remained stable at a high level.
		The cure rate remained stable at a high level.

## Data Availability

The data presented in this study are available on request from the corresponding author. The data are not publicly available because research is ongoing.
